# Melanocortin 4 Receptors in the Paraventricular Nucleus Modulate the Adipose Afferent Reflex in Rat

**DOI:** 10.1371/journal.pone.0080295

**Published:** 2013-11-11

**Authors:** Peng Li, Hai-Jian Sun, Ling-Li Zhang, Lei Ding, Ying Han, Guo-Qing Zhu, Ye-Bo Zhou

**Affiliations:** 1 Key Laboratory of Cardiovascular Disease and Molecular Intervention, Department of Physiology, Nanjing Medical University, Nanjing, China; 2 Department of Cardiology, the First Affiliated Hospital of Nanjing Medical University, Nanjing, China; Max-Delbrück Center for Molecular Medicine (MDC), Germany

## Abstract

**Background and Aim:**

Paraventricular nucleus (PVN) of hypothalamus is an important central component in modulating adipose afferent reflex (AAR). Melanocortin receptors (MC3/4Rs) expressions are found in the hypothalamic PVN. This study was designed to determine the roles of MC3/4Rs in the PVN in modulating the AAR and its downstream signaling pathway in normal rats.

**Methodology/Principal Findings:**

Renal sympathetic nerve activity (RSNA) and mean arterial pressure (MAP) were recorded in anaesthetized rats. AAR was evaluated using RSNA and MAP responses to capsaicin injection into the inguinal white adipose tissue (iWAT). Microinjection of the MC3/4R agonist melanotan II (MTII) into the PVN enhanced the AAR. The MC3/4R antagonist SHU9119 or MC4R antagonist HS024 attenuated the AAR and abolished MTII-induced AAR response. The adenylate cyclase (AC) inhibitor SQ22536 or the protein kinase A (PKA) inhibitor Rp-cAMP attenuated the AAR and the effect of MTII on the AAR was abolished by pretreatment with SQ22536 or Rp-cAMP in the PVN. Furthermore, both PVN microinjection of MTII and iWAT injection of capsaicin increased the cAMP level in the PVN. SHU9119 in the PVN abolished the increase in cAMP level which induced by iWAT injection of capsaicin.

**Conclusion:**

The activation of MC4Rs rather than MC3Rs enhances the AAR, and a cAMP-PKA pathway is involved in the effects of MC4Rs in the PVN.

## Introduction

It has been reported that unilateral injection of leptin into the epididymal white adipose tissue (eWAT) reflexly increases the efferent activity of the sympathetic nerve innervating the bilateral eWAT [1], the brown adipose tissue (BAT), adrenal medulla, pancreas, liver and reduces the vagus nerve activity [2]. Anterograde transneuronal viral tract tracing reveals central sensory circuits from WAT [3]. Chemical stimulation of WAT-induced sympatho-excitatory reflex is called adipose afferent reflex (AAR), we recently found that WAT injection of capsaicin, adenosine, bradykinin, or leptin increased renal sympathetic nerve activity (RSNA) and mean arterial pressure (MAP) in rats. Furthermore, WAT injection of capsaicin increased afferent and efferent nerve activity of the WAT [4]. 

Paraventricular nucleus (PVN) of hypothalamus is an important central component in modulating the chemoreflex [5], baroreflex [6,7], cardiac sympathetic afferent reflex [8] and AAR [4]. It plays a key role in regulating the sympathetic and cardiovascular activities via its projections to the rostral ventrolateral medulla and intermediolateral column of the spinal cord [9]. Melanocortins (MCs) are peptides derived by proteolytic cleavage from pro-opiomelanocortin, and include α-, β- and γ-melanocyte-stimulating hormones (MSHs) and adrenocorticotropic hormone (ACTH). Five types of melanocortin receptors (MCRs) have been identified, of which moderate MC3R and high MC4R expressions are found in the hypothalamic PVN [10-12]. Our previous study has shown that the activation of MC3/4Rs in the PVN increases the sympathetic outflow and blood pressure (BP) [13]. It has been found that a high-fat diet increases endogenous activity of the central MC3/4Rs and that MC3/4Rs appear to play an important role in linking increased BP with diet-induced obesity [14]. However, it is unknown whether MC3/4Rs in the PVN are involved in the regulation of AAR. 

Our study has demonstrated that MC3/4Rs in the PVN increases the sympathetic outflow and BP via the cAMP–protein kinase A (PKA) pathway [13]. It has been reported that MC3/4R agonist MTII rapidly increases the phosphorylation of both extracellular signal-regulated kinase 1/2 and cAMP response element-binding protein in a dose-dependent manner in the nucleus tractus solitarii [15]. Pharmacological blockade of tropomyosin-related kinase receptor type B (TrkB) abolished the anorexigenic effect of a selective MC4R agonist [16]. However, the signaling pathway of melanocortin involved in the AAR is not well elucidated.

The present study was designed to determine whether MC3/4Rs in the PVN are involved in modulating AAR and the mechanism of its action. 

## Materials and Methods

Experiments were carried out on male Sprague-Dawley rats weighing between 350 and 400 g. The procedures were approved by the Experimental Animal Care and Use Committee of Nanjing Medical University and complied with the Guide for the Care and Use of Laboratory Animals (the 8th edition, 2011). All efforts were made to minimize the number of animals used and their suffering. The rat was anesthetized by intraperitoneal injection of urethane (800mg kg^-1^) and α-chloralose (40 mg kg^-1^) and ventilated with room air using a rodent ventilator (683, Harvard Apparatus). The right carotid artery of rat was cannulated for recording arterial blood pressure (ABP).

### RSNA Recording

RSNA used to evaluate the dynamic changes of sympathetic outflow. The left renal sympathetic nerve was isolated through a retroperitoneal incision and cut distally, then placed on a pair of silver recording electrodes. By using an AC/DC differential amplifier (DP-304, Warner Instruments, Hamden, CT, USA), the signals of the nerve were amplified with a low-frequency cutoff at 10 Hz and a high-frequency cutoff at 3000 Hz. The amplified and filtered signals were integrated at time constant of 1.0 s. The raw and the integrated RSNA were simultaneously recorded on a PowerLab data acquisition system (8SP; AD Instruments). The percentage change in integrated RSNA from the baseline value was calculated after intervention. 

### Microinjection into PVN

**Figure 1:**
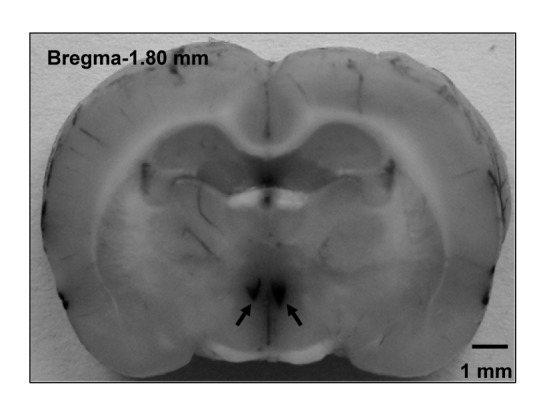
A representative coronal brain slice of rat shows the sites of the PVN microinjection. Arrows point to the microinjection sites in the PVN.

The rats were placed in a stereotaxic frame (Stoelting, Chicago, IL, USA). According to the stereotaxic atlas of Paxinos & Watson (2005), the coordinates for the PVN are 1.8 mm caudal to bregma, 0.4 mm lateral to the midline and 7.9 mm ventral to the dorsal surface. The bilateral PVN microinjections were carried out with two glass micropipettes (about 50 μm tip diameter) and finished within 1 min (50 nl for each side of the PVN). To exclude the possibility that the effects of MTII were caused by diffusion to other brain area, the effects of microinjection of MTII (0.2 nmol) into the anterior hypothalamic area which is adjacent to the PVN were determined in anesthetized rats (n= 6). The coordinates for the anterior hypothalamic area are 1.8 mm caudal to bregma, 1.0 mm lateral to the midline and 9.0 mm ventral to the dorsal surface. At the end of each acute experiment, the same volume of Evans Blue (2%) for histological identification was injected into the microinjection site in rats. The microinjection sites became deep blue after Evans Blue injection through observing the coronal sections at the PVN level with naked eye. A representative coronal brain slice of rat shows the sites of the PVN microinjection (Figure 1). The microinjection sites used for data analysis were within the marginal regions of the PVN. The rat was excluded from data analysis if the distance between the centre point of microinjection and the boundary of the PVN was less than 0.15 mm. In order to avoid disturbing the results of enzyme linked immunosorbent assay (ELISA), the rats used for the cAMP measurement did not receive Evans Blue (2%) injection. 

### Evaluation of AAR

The AAR was induced as previously reported [4]. The right inguinal WAT (iWAT) was exposed through an inguinal area incision. Four thin and sharp stainless steel tubes (outer diameter: 0.31 mm) were inserted into the fat pad 3 mm below the surface of the fat pad. The tips of these tubes were 4 mm apart from each other and were connected with a 4-channel programmable pressure injector (PM2000B, MicroData Instrument, NJ, USA). The AAR was induced by the injections of capsaicin (1.0 nmol μl^-1^) into four sites of the right iWAT at a rate of 4.0 μl min^-1^ for 2 min. AAR was evaluated by the RSNA and MAP responses to capsaicin injections. At the end of the experiment, the same volume of Evans blue dye was injected into the iWAT for 30 min for histological identification, and the dye was localized in the WAT and the diffusion diameter was less than 3 mm in all rats.

### Measurement of cAMP Level in the PVN

The rats were killed with an overdose of intravenous pentobarbital injection (100 mg kg^−1^). The brains were removed and quickly frozen in liquid nitrogen and stored at −80 °C until being sectioned. Coronal sections of the brain were made with a cryostat microtome (Leica CM1900-1-1, Wetzlar, Hessen, Germany) at the PVN level according to the atlas of Paxinos & Watson (2005). The thickness of the PVN sections used for cAMP analysis was 450 μm. The PVN areas were punched out and homogenized in lysis buffer. The total protein in the homogenate was extracted and measured using a protein assay kit (BCA; Pierce, Santa Cruz, CA, USA). The cAMP levels of the PVN were determined by an enzyme immunoassay kit (Cayman Chemical Co, USA) following the manufacturer’s instructions.

### Chemicals

Melanotan II (MTII, an MC3/4R agonist), Ac-Nle-c(Asp-His-D-2-Nal-Arg-Trp-Lys)-NH_2_ (SHU9119, an MC3/4R antagonist) and cyclic (AcCys^3^,Nle^4^,Arg^5^,D-Nal^7^,Cys-NH_2_^11^) α-MSH-(3-11) (HS024, a selective MC4R antagonist) were from Bachem (Bubendorf, Switzerland). Dibutyryl-cAMP (db-cAMP, a cAMP analogue), 9-(tetrahydro-2-furanyl)-9H-purin- 6-amine (SQ22536, an adenylyl cyclase inhibitor), rp-adenosine-3',5'-cyclic monophosphorothionate(Rp-cAMP, a PKA inhibitor) and capsaicin were from Sigma Chemical Co. (St Louis,MO, USA). Capsaicin stock solution was dissolved in absolute ethanol and was diluted before injection to a final concentration of 1% of the stock solution, 1% of Tween 80, and 98% of normal saline. Other chemicals were dissolved in normal saline. 

### Experimental Protocols

#### Protocol 1.

The effect of MTII (0.1, 0.2 or 0.4 nmol) in the PVN on the AAR was determined in anesthetized rats. Chemical microinjection into the PVN was finished within 1 min prior to the induction of AAR respectively. The AAR was evaluated 8 min after the chemicals microinjection into the PVN. The changes of RSNA and MAP responses to capsaicin injection into the iWAT were determined by averaging 2 min of the maximal responses and compared with the values before the microinjection (n = 6 for each group). 

#### Protocol 2.

The mechanism of MTII in the PVN in AAR was explored in anesthetized rats. At first, the effect of MC3/4R antagonist SHU9119 (0.4 nmol) or selective MC4R antagonist HS024 (1.0 nmol) in the PVN on the AAR was determined respectively, then pretreatment with PVN microinjection of saline, SHU9119 or HS024 on the AAR response to MTII was investigated respectively. The pretreatment was performed 8 min before MTII treatment in the PVN, and the AAR was evaluated 8 min after MTII. For the rats in pretreatment groups, the RSNA and MAP changes induced by MTII were determined by averaging 2 min of the maximal responses to MTII and compared with the values before MTII (n = 6 for each group). 

#### Protocol 3.

The signaling pathway of MTII involved in the AAR in the PVN was explored in anesthetized rats. At first, the effect of cAMP analogue db-cAMP (1.0 nmol), adenylyl cyclase (AC) inhibitor SQ22536 (2.0 nmol) or PKA inhibitor Rp-cAMP (1.0 nmol) in the PVN on the AAR was determined respectively, then pretreatment with PVN microinjection of saline, db-cAMP, SQ22536 or Rp-cAMP on the AAR response to MTII was investigated respectively. The pretreatment was performed 8 min before MTII treatment in the PVN, and the AAR was evaluated 8 min after MTII. For the rats in pretreatment groups, the RSNA and MAP changes induced by MTII were determined by averaging 2 min of the maximal responses to MTII and compared with the values before MTII (n = 6 for each group). 

#### Protocol 4.

In order to further clarify whether the cAMP-PKA pathway was involved in the effect of MC4Rs in the PVN on the AAR, the cAMP level in the PVN was determined in anesthetized rats. Rats were decapitated 8 min after the PVN microinjection or 15 min after the iWAT injection. At first, the effect of PVN microinjection of saline or MTII (0.2 nmol) on cAMP level in the PVN was determined respectively, then the effect of iWAT injection of saline or capsaicin on cAMP level in the PVN was determined respectively, at last, the effect of the PVN pretreatment with saline or SHU9119 (0.4 nmol) on cAMP level induced by iWAT injection of capsaicin was determined respectively (n = 6 for each group).

### Statistical Analysis

All data are expressed as means ± SE. Comparisons between control and experimental interventions were made using Student’s t-test. One-way ANOVA was used, followed by Bonferroni test for post hoc analysis when multiple comparisons were made. A linear regression analysis was made to determine the correlation between the doses of MTII and the RSNA or MAP changes. A value of P< 0.05 was considered statistically significant.

## Results

### Dose Effects of MC3/4R Agonist in the PVN

**Figure 2:**
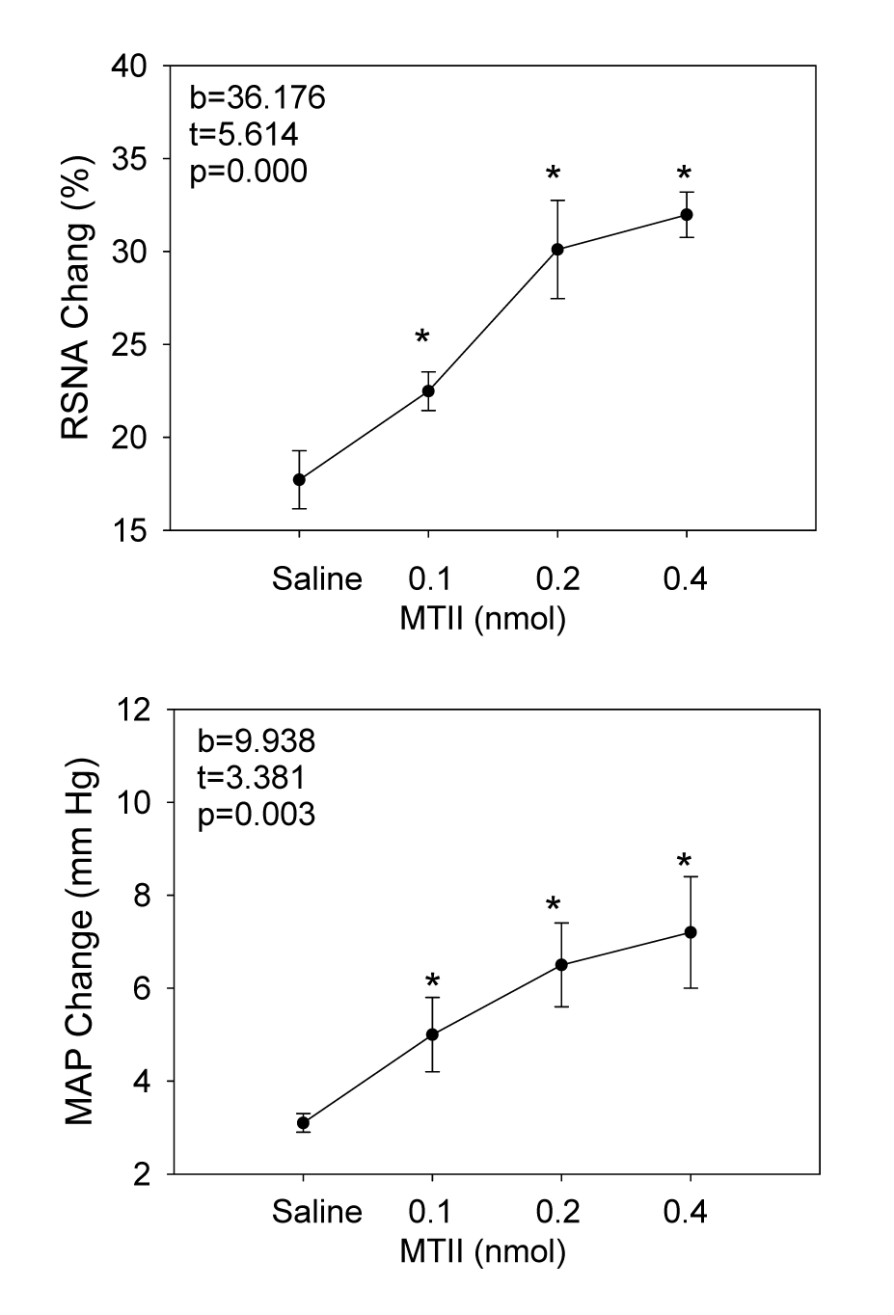
The effect of the PVN microinjection of saline or three doses of MTII (0.1, 0.2 or 0.4 nmol) on the AAR. The MTII doses 0.1, 0.2 and 0.4 nmol represent the total dose injected into both sides of the PVN. The AAR was evaluated by the RSNA and MAP responses to capsaicin injection into the inguinal white adipose tissue (iWAT). The total dose of capsaicin was 32 nmol in a rat. Values are Mean±SE. *P<0.05 versus Saline. n = 6 for each group. Abbreviations: b, β coefficient (standardized regression coefficient); t, *t* value, which is used to test the significance of the regression coefficient; RSNA, renal sympathetic nerve activity; MAP, mean arterial pressure.

**Figure 3:**
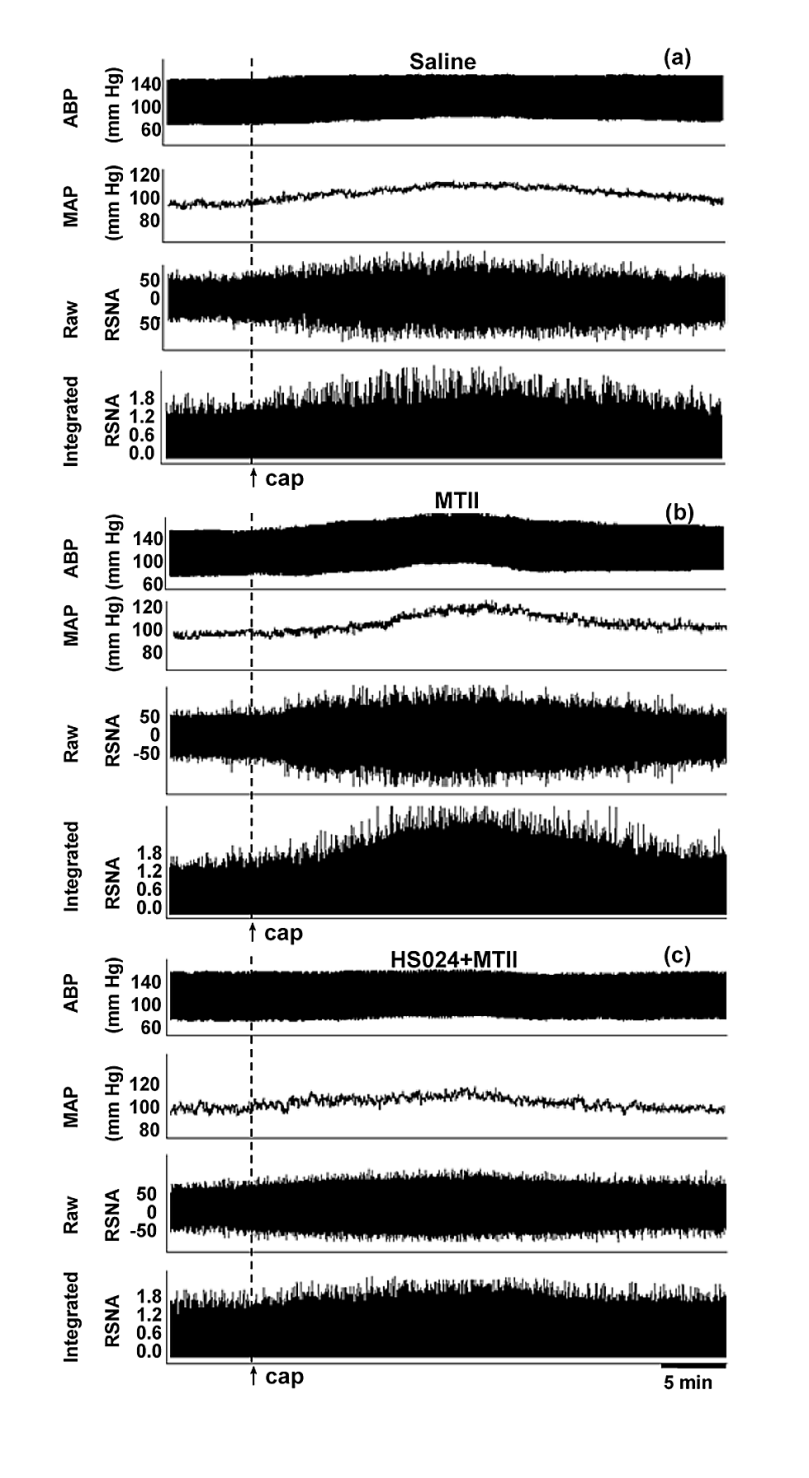
Representative recordings showing the adipose afferent reflex (AAR). The effect of microinjection of saline (a), MTII (0.2 nmol) (b) into the PVN on the AAR and the effect of PVN microinjection of MTII on the AAR after pretreatment with HS024 (1.0 nmol) (c). Abbreviation: ABP, arterial blood pressure; MAP, mean arterial pressure.

Microinjection of three doses of MC3/4R agonist MTII (0.1, 0.2 or 0.4 nmol) significantly enhanced the AAR in a dose-dependent manner (Figure 2). There was a significant positive correlation between the dose of MTII and the AAR (Figure 2). The effects of MTII peaked at about 15-20 min and lasted at least 30 min (Figure 3b). Injection of capsaicin (1.0 nmol μl^-1^) into the iWAT significantly increased the RSNA in the period of 5–25 min and MAP in the period of 5–20 min but had no significant effect on the HR. The RSNA change reached its maximum of ~17% at 13 min or so, whereas the MAP change reached its maximum of ~ 3 mmHg at 8 min or so. The representative recordings showed that the AAR was induced by injection of capsaicin (1.0 nmol μl^-1^) into the iWAT (Figure 3a) and PVN microinjection of MTII (0.2 nmol) enhanced the AAR (Figure 3b). In six rats, microinjection of same dose of MTII into the anterior hypothalamic area, which is adjacent to the PVN, had no significant effects on the changes of the RSNA and MAP induced by injection of capsaicin into the iWAT, data not shown.

### Effects of MC3/4R Antagonists in the PVN

**Figure 4:**
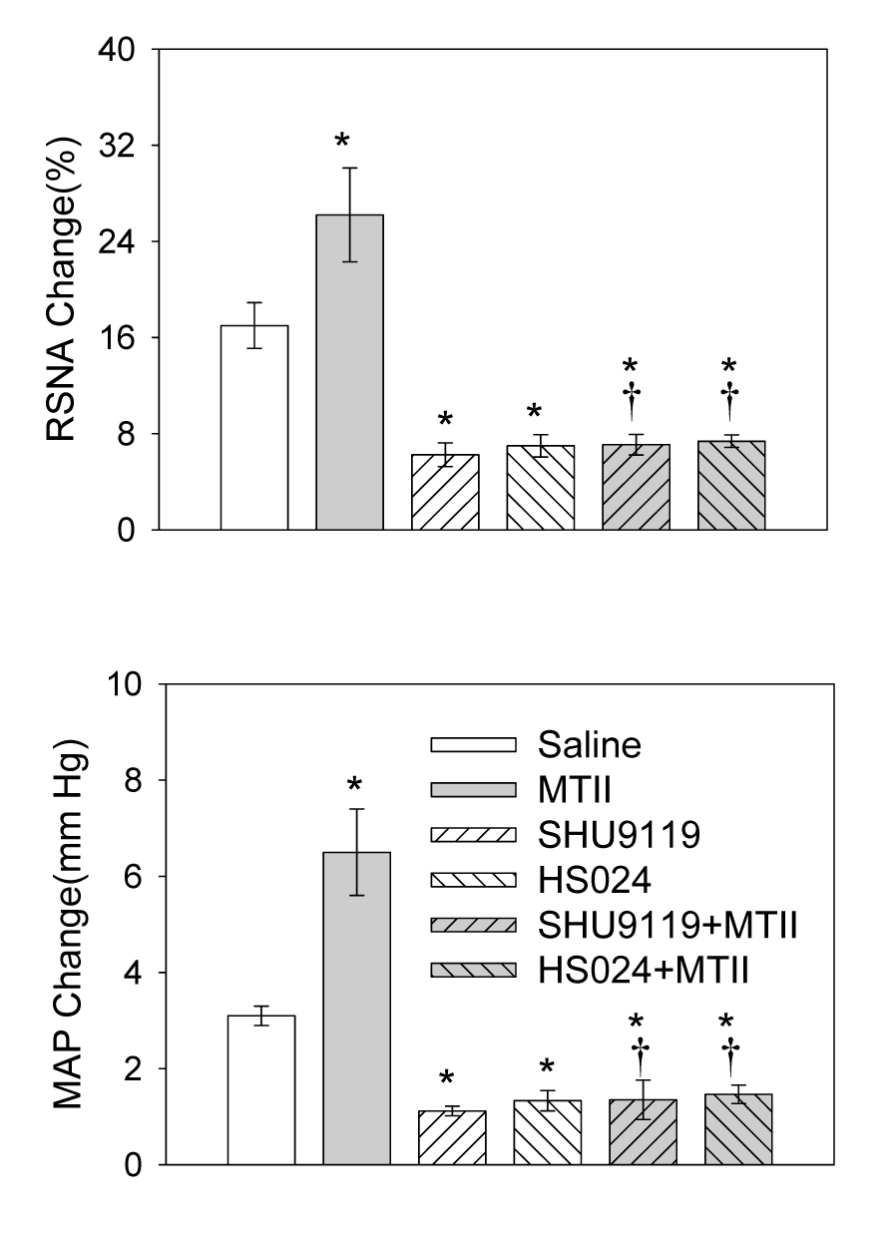
The effect of PVN microinjection of saline, MTII (0.2 nmol), SHU9119 (0.4 nmol) or HS024 (1.0 nmol) on the adipose afferent reflex (AAR) and the effect of PVN microinjection of MTII on the AAR after pretreatment with SHU9119 or HS024. MTII was administered 8 min after the microinjection of the MC3/4R antagonist SHU9119 or MC4R antagonist HS024. The AAR was evaluated by the RSNA and MAP responses to capsaicin application in the inguinal white adipose tissue (iWAT). Values are Mean±SE. *P<0.05 compared with Saline. †P<0.05 compared with MTII. n=6 for each group.

Microinjection of the MC3/4R antagonist SHU9119 or MC4R antagonist HS024 for 8 min significantly attenuated the AAR. Pretreatment with SHU9119 abolished the AAR enhancement response to MTII. MC4R antagonist HS024 almost produced the same effects as the MC3/4R antagonist SHU9119 (Figure 4). The representative recording showed that pretreatment with HS024 (1.0 nmol) abolished the AAR enhancement responses to MTII (Figure 3c). 

### Effects of cAMP Analogue or AC Inhibitor in the PVN

**Figure 5:**
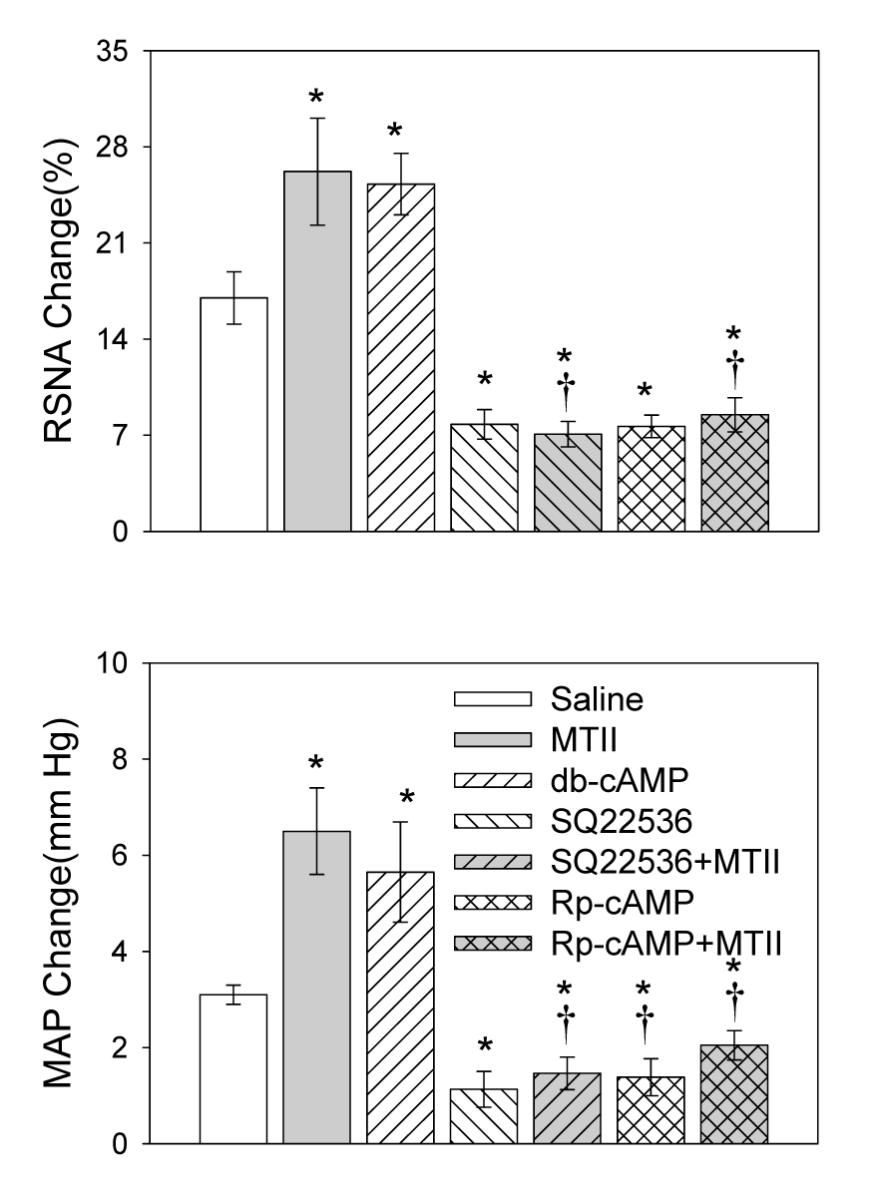
The effect of PVN microinjection of the cAMP analogue db-cAMP (1.0 nmol), the AC inhibitor SQ22536 (2.0 nmol) or the protein kinase A (PKA) inhibitor Rp-cAMP (1.0 nmol) and MTII after pretreatment with SQ22536 or Rp-cAMP on the adipose afferent reflex (AAR). Melanotan II was administered 8 min after the microinjection of SQ22536 or Rp-cAMP. The AAR was evaluated by the RSNA and MAP responses to capsaicin application in the inguinal white adipose tissue (iWAT). Values are Mean±SE. *P<0.05 compared with Saline. †P<0.05 compared with MTII. n=6 for each group.

PVN microinjection of db-cAMP, a cAMP analogue, enhanced the AAR. PVN microinjection of SQ22536, an AC inhibitor, attenuated the AAR and abolished MTII-induced AAR enhancement response (Figure 5).

### Effects of PKA Inhibitor in the PVN

PVN microinjection of Rp-cAMP, a PKA inhibitor, attenuated the AAR and abolished MTII-induced AAR enhancement responses (Figure 5).

### Determination of cAMP Level in the PVN

**Figure 6:**
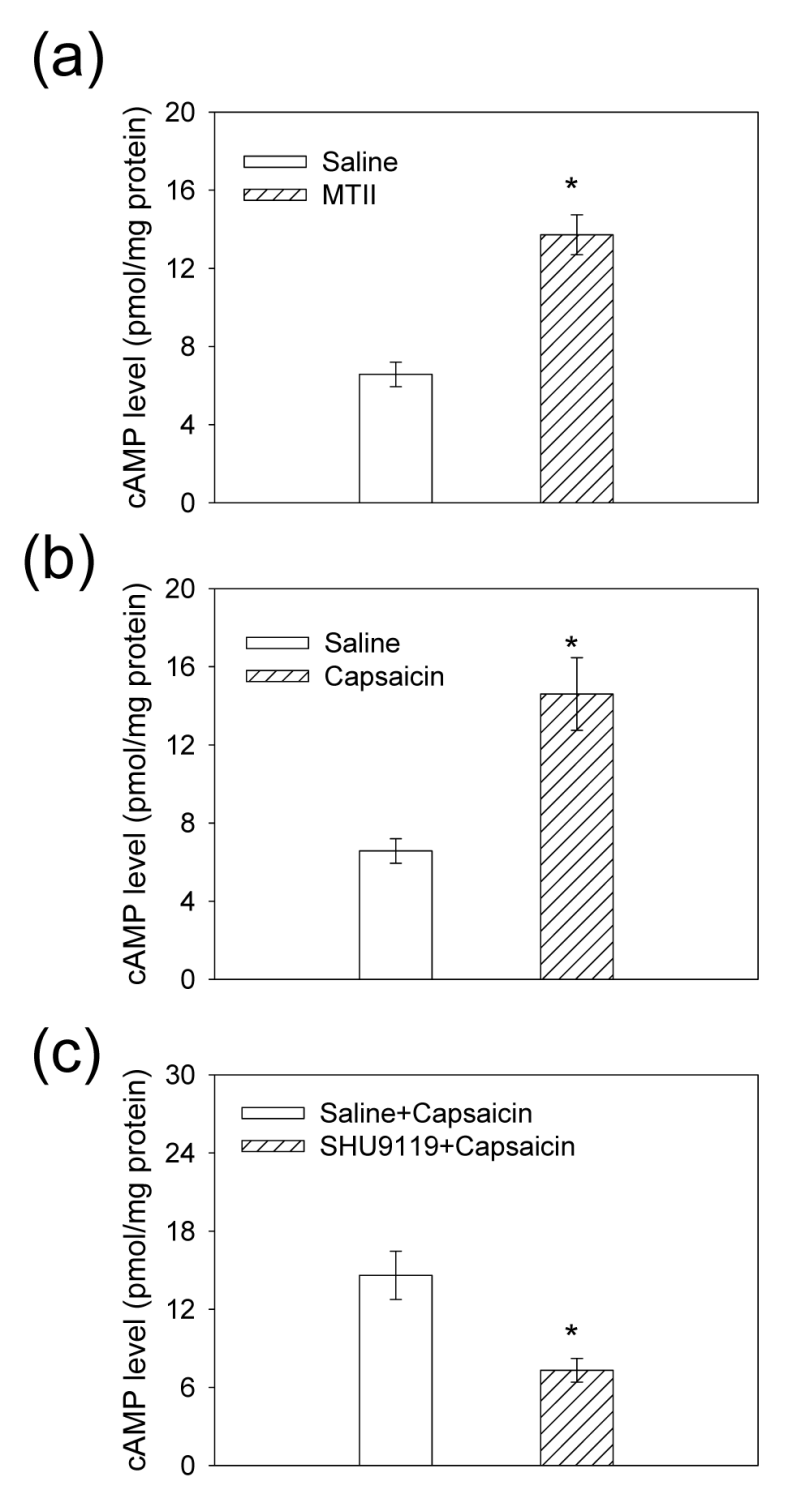
The cAMP level in the PVN under different conditions. The effect of the PVN microinjection of saline or MTII (0.2 nmol) (a) and iWAT application of saline or capsaicin (b) on the cAMP level in the PVN. The effect of pretreatment with saline or SHU9119 (0.4 nmol) in the PVN on the cAMP level response to iWAT injection of capsaicin (c). Values are Mean±SE. *P<0.05 versus Saline. n = 6 for each group.

Compared with the saline, both PVN microinjection of MTII and iWAT injection of capsaicin caused greater increase in cAMP level in the PVN (Figure 6a, 6b), PVN pretreatment with SHU9119 decreased cAMP level following iWAT capsaicin injection (Figure 6c).

## Discussion

Previous studies from our laboratory demonstrated that AAR induced by WAT injection chemicals such as capsaicin, bradykinin, adenosine and leptin resulted in the increases of sympathetic outflow and BP [4]. Capsaicin causes neuronal excitation, low concentration of capsaicin is usually used to determine the function of sensory afferents [17-19]. Bradykinin, leptin, or adenosine in the iWAT caused similar effects on RSNA and MAP as capsaicin [4,20]. Hypothalamic PVN is an important central site involved in the regulation of AAR [4,20]. Enhanced AAR activates sympathetic nervous system and increases circulating renin, angiotensin II (Ang II), and norepinephrine (NE) levels in the obesity hypertensive rats [20]. The present study demonstrates new findings that in the PVN, MC4Rs enhance the AAR and a cAMP-PKA signaling pathway participates in MC4R-mediated effects. 

The MC3/4Rs are expressed in the PVN [10-12]. MTII in the PVN increased sympathetic nerve activity, which was mediated by MC3/4R activation. Endogenous MC3Rs in the PVN exerts a tonic excitatory effect on sympathetic activity [13]. MC3/4R agonist MTII had a long-lasting inhibitory effect on the food intake [21]. Central MC3/4Rs are also known to participate in the regulation of energy metabolism and food intake. MC3R and MC4R knockout mice exhibited obese phenotypes [22]. Intracerebroventricular (icv) administration of MC4R agonist produced a dose-dependent increase in sympathetic nerve activity to brown adipose tissue [23]. Neuroanatomical and functional evidences demonstrated that MC4R mRNA expressed in the PVN neurons involved in sympathetic outflow not only to the white adipose tissue but also to the brown adipose tissue [24,25]. So it is speculated that MC4Rs in the PVN could be involved in the regulation of AAR.

Icv infusion of MC3/4R antagonist SHU9119 reduced MAP in the rats [26]. HS024, a cyclic MSH analogue, is a potent and selective MC4R antagonist [27]. In the present study, MTII in the PVN enhanced the AAR, selective MC4R antagonist HS024 attenuated the AAR and abolished MTII-induced AAR enhancement response, which was similar with the effects of SHU9119. These results suggest that the activation of MC4Rs rather than MC3Rs in the PVN mainly enhances the AAR. The effect of SHU9119 (MC3/4R antagonist) attenuated the AAR by about 50%, which suggest that MC4Rs are partially involved in the AAR. Unfortunately, a selective MC3R antagonist is not available to date. One mechanism leading to the enhanced AAR caused by MTII microinjection into the PVN maybe the increased central gain of the reflex through the activation of MC4Rs in rats, so the activation of MC4Rs by interacting with MTII in the PVN were partially responsible for the increased central gain of AAR in rats.

It has been reported that MC4Rs regulates hippocampal synaptic plasticity through PKA dependent mechanism [28]. Activation of the MC4Rs induced the expression of brain derived neurotrophic factor in rat cultured astrocytes through cAMP–PKA pathway [29]. However, whether the cAMP-PKA pathway in the PVN is involved in MC4Rs-mediated enhancement of AAR is unknown. Exposure of Down’s syndrome human fetal skin fibroblasts to db-cAMP (a cAMP analogue) stimulated PKA activity [30]. In the present study, microinjection both SQ22536 (a specific membrane-permeable AC inhibitor) and Rp-cAMP (a specific membrane-permeable PKA inhibitor) [31-33] into the PVN reduced the AAR and abolished the effects of MTII-induced AAR enhancement. Furthermore, both PVN MTII microinjection and iWAT capsaicin injection caused a marked increase in cAMP level in the PVN. PVN pretreated with SHU9119 abolished iWAT injection of capsaicin-induced cAMP level increase in the PVN. These results indicate that the cAMP-PKA pathway in the PVN mediates the effect of MC4Rs on the AAR. 

Central melanocortins are involved in hypertension and obesity. Chronic central administration of the MC3/4R antagonist SHU9119 caused a greater reduction in BP in spontaneously hypertensive rats than in Wistar–Kyoto rats despite marked increases in food intake, weight gain and insulin resistance [34]. A possible physiological significance of the AAR is to regulate metabolism of the adipose tissue by increasing sympathetic nerve activity, which may promote the decrease in body weight and increase in energy expenditure as a negative feedback effect [1,2]. However, the enhanced AAR contributes to sympathetic activation in diet-induced obesity hypertension [20]. Persistent increase in afferent signals from WAT in overweight and obesity will result in excessive sympathetic activation which increases peripheral resistance and contributes to the development of hypertension and related organ damage. Therefore, depressing the enhancement of AAR in the PVN may be a very good strategy to decrease sympathetic activation and BP in obesity hypertension by blocking the activation of MC4Rs.

In the present study, we found that the activation of MC4Rs in the PVN by MTII increased the AAR caused by iWAT injection of capsaicin; MC4Rs antagonist, AC or PKA inhibitors obviously inhibited the AAR and abolished the enhancement of AAR induced by MTII; PVN microinjection of MTII or iWAT injection of capsaicin increased the cAMP level in the PVN and PVN pretreatment with MC4Rs antagonist reduced the cAMP level response to capsaicin injection into the iWAT. In conclusion, MC4Rs participated in the regulation of AAR via receptor activated cAMP-PKA pathway, and endogenous MC4Rs rather than MC3Rs in the PVN play an important role in the AAR. All functional experiments of MTII in the PVN about AAR were performed under anesthetized rats, the physiological roles of MTII and its related mechanisms in AAR need to be further investigated in conscious rats in the future.
